# Investigating the environmental Kuznets curve between economic growth and chemical fertilizer surpluses in China: a provincial panel cointegration approach

**DOI:** 10.1007/s11356-021-17122-0

**Published:** 2021-10-23

**Authors:** Xiaomin Yu, Karsten Schweikert, Reiner Doluschitz

**Affiliations:** 1 Institute of Farm Management, University of Hohenheim, 70593 Stuttgart, Germany; 2 Core Facility Hohenheim & Institute of Economics, University of Hohenheim, 70599 Stuttgart, Germany

**Keywords:** Chemical fertilizer surplus, China, EKC, Cointegrating panel regression, Regional

## Abstract

This study investigated the relationship between fertilizer nitrogen (N) and phosphate (P) surpluses and economic development on the regional level in China. With a balanced panel dataset covering 30 provinces of mainland China from 1988 to 2019, we employed panel cointegrating polynomial regression (CPR) analysis using fully modified OLS (FM-OLS) estimators. Our results suggested that all provinces exhibit a long-run cointegrated relationship between fertilizer surpluses and real per capita gross regional product (GRP). A total of 22 provinces out of 30 showed a significant inverted U-shaped environmental Kuznets curve (EKC). Among those, 14 provinces are considered to have reached the peak and 8 provinces are considered to be before the peak. The group-mean turning points on the EKC are CNY 7022, CNY 9726, CNY 4697, CNY 3749, and CNY 5588 per capita GRP (1978 = 100) for the Northeast, Northcentral, Middle, and lower reaches of the Yangtze River, Southwest and Northwest China, respectively. The overall turning point of China is CNY 6705 per capita real gross domestic product (GDP), which was reached in circa 2012. This shows a general improvement of chemical fertilizer management in China. However, six provinces still exhibit linear growth in fertilizer surpluses when the economy grows. These regions are characterized by high cash-crop ratios and are mostly located along the southeast coast. Therefore, more effort and attention should be given to these regions to promote further fertilizer reduction. At the same time, nutrient use efficiencies should be improved, especially for cash crops such as fruit and vegetables.

## Introduction

Chemical fertilizer use has been an essential component in modern agricultural production, comprising the majority of plant nutrients to sustain the current crop yields and soil fertility (Tilman et al. 2002; Stewart et al. 2005). An evaluation of long-term studies showed that fertilizer inputs accounted for 40 to 60% of crop yields in temperate climates, and for even higher proportions in the tropics (Stewart and Roberts 2012). On the one hand, this has substantially contributed to hunger reduction worldwide and the sustainment of food security (Bruinsma 2003; Bindraban et al. 2020). On the other hand, the excessive use of fertilizers in recent decades has not only led to its diminishing returns in yield improvement, but also caused numerous environmental problems, such as water impairment, soil acidification, and air pollution (Tilman et al. 2002; Gruber and Galloway 2008; Parris 2011; Savci 2012).

**Fig. 1:**
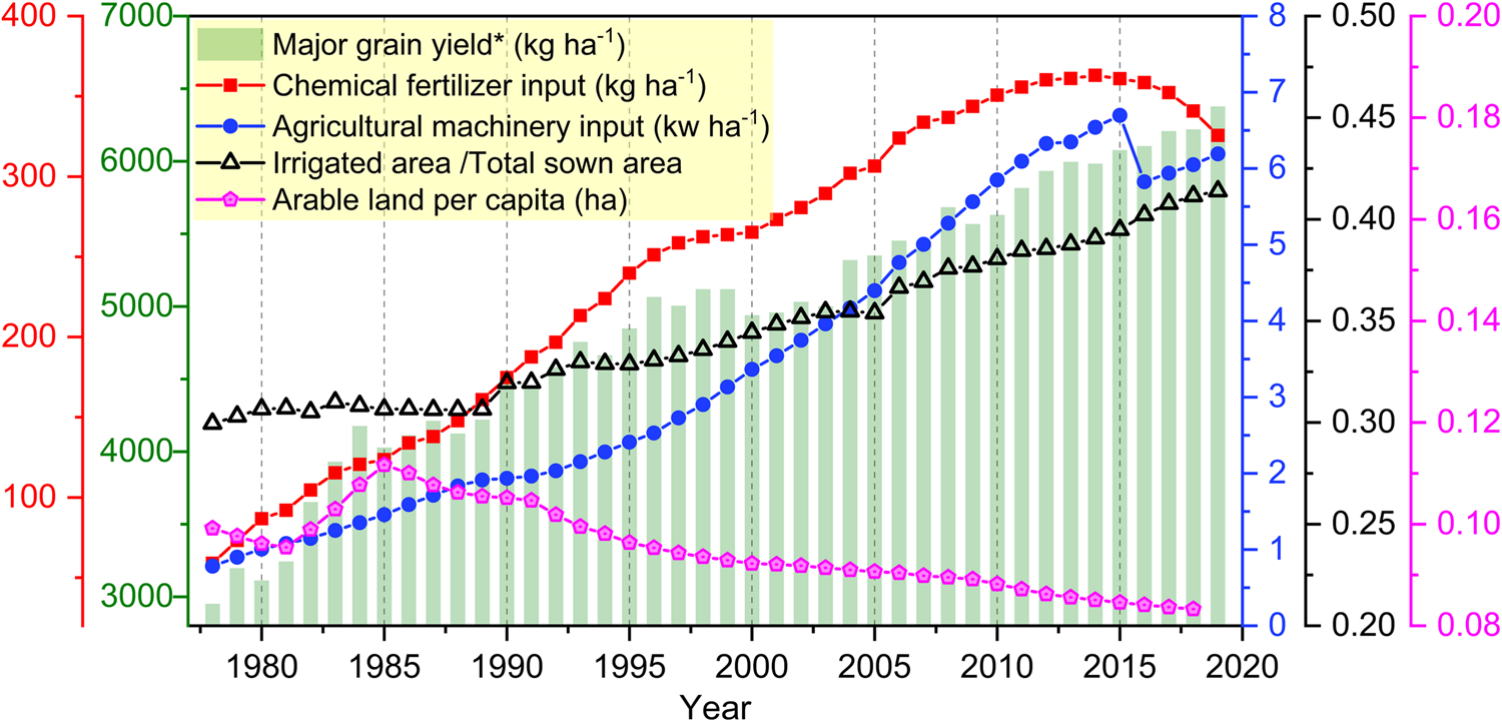
This figure displays average yields of major grains and major agricultural inputs in China from 1978 to 2019. *Major grains here include rice, wheat, and maize. Source of data: NBS and FAOSTAT

The fertilizer dilemma — food or the environment — is especially pronounced in the case of China. In recent decades, China has experienced a significant expansion in agricultural productivity. While per capita arable land kept decreasing, the average major grain yields (wheat, rice, and maize) more than doubled from 2952 kg ha^−1^ in 1978 to 6378 kg ha^−1^ in 2019 (NBS 2020). At the same time, China’s value added in agriculture, forestry, and fishery increased 25-fold and reached USD 1.02 trillion in 2019 (The World Bank 2021). Significant factors contributing to the agricultural productivity boost are the drastically intensified agricultural inputs (Fig. 1). Over the last four decades, China’s per hector inputs of chemical fertilizer and agricultural machinery increased 5.5-fold and eightfold, respectively. The ratio of sown area under irrigation also grew from 0.30 in 1978 to 0.41 in 2019 (NBS 2020). In 2014, China’s chemical fertilizer use per sown area reached its peak (363 kg ha^−1^), which is three times the world’s average (121 kg ha^−1^) and more than twofold that of the US and the European Union (FAOSTAT 2020; NBS 2020).

Coupled with China’s excessive use of chemical fertilizers, in recent years, there have been reports of massive agricultural non-point source pollution (Ongley et al. 2010; Zhao et al. 2011; Sun et al. 2012; Smith and Siciliano 2015). To cope with this, in 2015, the Chinese Ministry of Agriculture developed the Zero Growth Action Plans for fertilizer and pesticide use by 2020 (MoA 2015; Jin and Zhou 2018). Since then, an overall reduction of fertilizer consumption in China has been observed, although regional variations still persist (Jin et al. 2018, 2019; Yu et al. 2020).

Introduced by the pioneering work of Grossman and Krueger (1991, 1995) and Panayotou (1995), the environmental Kuznets curve (EKC) theory has been widely discussed in recent decades in studies investigating the relationship between environmental pollution and economic growth. The EKC theory implies that, as the economy develops, the environmental degradation first increases and, when a certain income level is reached, decreases again. Therefore, the relationship between the environmental and economic indicator appears as an inverted U-shaped curve. Our objective is to analyze the relationship between chemical fertilizer surpluses and economic growth on the regional level in China as well as to test the existence of an inverted U-shaped EKC between the two indicators. The chemical fertilizer surpluses here are defined as the positive difference between fertilizer nitrogen (N) and phosphate (P) inputs and their outputs (Liu et al. 2010; Bouwman et al. 2013). We collected a balanced panel dataset covering 30 provinces of mainland China with a sampling period from 1988 to 2019. Panel cointegrating polynomial regressions (CPR) (Wagner and Hong 2016; Wagner and Reichold 2018) were estimated using fully modified OLS (FM-OLS) to study the EKC relationship.

Our study contributes to the existing literature in multiple ways. Firstly, we explicitly analyzed time-series fertilizer N and P surpluses for each province of China and used them as the environmental indicators instead of using the fertilizer application rates. This helped to exclude confounding factors related to the inhomogeneity of nutrient use efficiencies and soil conditions of different regions. Secondly, in contrast to existing studies, we applied state-of-the-art methods to model our nonstationary variables, namely estimating CPRs with appropriate FM-OLS estimators (Wagner 2015; Wagner and Hong 2016), to provide valid inferential results. Finally, our analysis integrated the empirical results with, inter alia, regional socio-economic backgrounds and policy implications in China, providing a comprehensive analysis and overview of China’s chemical fertilizer use.

The present paper proceeds as follows. The “Literature review” section provides a literature review concerning agrochemical consumption in the context of the EKC hypothesis. The “Methodology” section introduces the theoretical framework, data, and methodology employed in the study while the “Results” section presents the results of the empirical analysis. The “Discussion” section discusses the results in the context of socio-economic development, policy interventions, and cropping structures in China. The “Conclusion” section draws conclusions, puts forward policy implications, and addresses limitations and further research directions of the study.

## Literature review

Although various pollution indicators have been applied in the EKC context, overwhelmingly, it has been greenhouse gas emissions that have been selected and investigated (e.g., Yang et al. 2017; Ali et al. 2017; Olale et al. 2018; Hanif et al. 2019; Murshed et al. 2020; Anwar et al. 2021; Alharthi et al. 2021). Sarkodie and Strezov (2019) categorized the indicators used in the majority of EKC-related studies into four categories, namely atmospheric indicators; land indicators; oceans, seas, coasts, and biodiversity indicators; and freshwater indicators. Their findings revealed that the current EKC-related studies were predominately based on atmospheric indicators, whereas studies concerned with other aspects were still limited.

### Previous studies concerning agrochemical use in the EKC context

Over the last decade, research investigating the relationship between economic development and agriculture-induced pollution has gained considerable attention (Liang 2016; Ali et al. 2017; Ridzuan et al. 2020; Selcuk et al. 2021). Among those, a few studies discussed agrochemical consumption (e.g., chemical fertilizers, pesticides, and agricultural plastic films) as a land indicator in the framework of the EKC hypothesis, recognizing them as a source of agricultural non-point pollution (see Tables 1 and 2 for an overview of the literature). Longo and York (2008) are some of the pioneers mentioning fertilizer and pesticide consumption in the EKC context. They employed cross-national data from the year 2000 to empirically investigate the relationship among structural factors, e.g., economic development, export intensity, and agrochemical consumption among nations using OLS regression models. Their results showed that, within the range of the observed GDP per capita, there was some indication of an EKC in terms of pesticide use but not fertilizer use.

**Table 1:** A collection of *cross-national* EKC-related studies using agrochemical use as the environmental indicator

Reference	Case study	Data period	Key variables	Methodology	Major results
(Celikkol Erbas and Guven Solakoglu 2017)	145 countries	2002–2010	N_2_O emission from N fertilizers, per capita GDP and the share of agricultural sector in GDP	Vector Error Correction Model (VECM)	A long-run causality between agricultural emissions and income, agriculture share, and land temperature anomalies was confirmed. A short-run EKC relationship was found between agricultural emissions and incomes of various countries
(Hedlund et al. 2020)	106 nations	1990–2014	Total pesticide consumption (incl. insecticide, herbicide, and fungicide), value added in agriculture, agricultural employment, arable land, food exports, GDP per capita, population	Fixed effects panel regression models	A positive relationship between economic growth and pesticide consumption over time was revealed. At higher levels of economic development, there would be no decline in pesticide use
(Longo and York 2008)	Cross-national	2000	Chemical fertilizer and pesticide use, agricultural exports, GDP per capita, irrigated land as a proportion of arable land, population	Ordinary least squares (OLS) regression models	While there is some indication of an EKC in terms of pesticide use, there is no indication of an EKC in relation to fertilizer use. The increases in exports of agricultural products contribute to increases in fertilizer and pesticide consumption within nations
(Pincheira et al. 2021)	177 countries	1973–2013	Planetary boundaries (incl. global chemical fertilizer consumption), and real GDP	OLS and fixed effect model, and the system generalized method of moment (GMM)	The existence of the classic EKC is supported for climate change and ocean acidification panels. However, biochemical cycles, ozone depletion and freshwater use, land change, and biodiversity loss boundaries do not support the EKC hypothesis using the same methodology
(Zhang et al. 2015)	113 countries	1961–2011	N surplus^a^ and GDP per capita	ADF unit root test and Autoregressive Distributed Lag (ARDL) (fixed effects model)	A significant quadratic relationship between GDP per capita and N surplus was revealed (*p* < 0.001). Regressions between GDP per capita and N surplus for each individual country fall into five response types: Bell shape, U-shape, linearly increase, not significant, and negative N surplus

**Table 2:** A collection of *China-specific* EKC-related studies using agrochemical use as the environmental indicator

Reference	Case study	Data period	Key variables	Methodology	Major results
(Chai et al. 2020)	Heilongjiang province, China	2005–2017	Application rates of chemical fertilizers, pesticides and agricultural films, and *farmers’ income*	Regression analysis	N-shaped relationships were found between both the application rate of chemical fertilizers and pesticide, and farmers’ income. The relationship between the consumption of agricultural films and farmers’ income followed an inverted U-shaped curve
(Gong and Tian 2010)^b^	China (average)	1978–2008	Application rate of chemical fertilizers, and per capita real *gross output value of crop production (GVC)*^c^	ADF unit root test and OLS regression	A significant inverted U-shaped EKC was observed between chemical fertilizer use and rural economic development in China. The per capita real GVC at the turning point was 547.67 CNY (1978 = 100), which has occurred in 2008
(Guo and Sun 2012)^b^	Jiangsu province, China	2001–2010	Application rates of chemical fertilizers and pesticides, density of livestock and poultry excrement, and real per capita GDP *of rural population*	ADF unit root test and non-linear regression analysis	Inverted U-shaped EKC were observed between agricultural pollution and rural economic development for the study area. GDP per capita at the turning points were 2130, 1970, and 3440 CNY for chemical fertilizers, pesticides, and livestock and poultry excrement, respectively
(Hong 2013)^b^	Chongqing municipality, China	1996–2010	Application rate of chemical fertilizers and *per capita real GVC*	OLS regression	There was a significant inverted U-shaped EKC between agriculture economic development and fertilizer input intensity. Turning point of the EKC occurred in 2009
(Li 2019)	Various provinces in China	1989–2017	N and P fertilizer use per capita (rural population), per capita real *gross output value of agriculture (GVA)*^c^, and per capita real GDP	Panel unit root test and panel cointegration	A significantly inverted U-shaped relationship was confirmed between economic growth variables and agricultural environmental pollution variables
(Li et al. 2016)	31 provinces in China	1989–2009	Fertilizer N and P surplus^a^, pesticide use intensity, and GDP per capita	Panel unit root, panel cointegration and panel-based dynamic OLS	A long-run cointegrated inverted U-shaped EKC was revealed between the environmental index and real GDP per capita. The value of the turning point is approximately 10,000–13,000, 85,000–89,000, and over 160,000 CNY, for synthetic fertilizer N indicator, fertilizer P indicator, and pesticide indicator, respectively
(Li and Zhang 2009)^b^	31 provinces in China	1998–2006	Application rates of chemical fertilizer and pesticide, density of livestock and poultry excrement, and per capita GDP	Random and fixed effects models	The inverted U-shaped EKC was confirmed between China’s agricultural non-point sources pollution and economic growth. Curves of all the three pollution sources are before the turning point
(Liu et al. 2009)^b^	31 provinces in China	1949–2007	Chemical fertilizer use and real *per capita GVA*	Non-linear regression analysis	27 provinces out of 31 had a significant relationship between fertilizer use and per capita GVA. 7 provinces had inverted U-shaped curves, while 10 had N-shaped and linear increase curves, respectively
(Liu et al. 2021a)	Three Gorges Reservoir region, China	2002–2017	Consumption of agrochemicals (chemical fertilizers, pesticides, and agricultural films), and per capita GDP	Spatial panel regression analysis	Inverted U-shaped EKCs were confirmed between economic development and chemical fertilizers and pesticides, respectively. Around half of the counties/districts have not met the corresponding inflection points of the EKCs
(Liu et al. 2021b)	Hubei province, China	2000–2017	Application rates of chemical fertilizers and the annual index of gross farming output	Unit root tests and panel regression analysis	An N-shaped EKC was confirmed between the annual county-specific fertilizer-impact indexes and the rural household income in Hubei, China
(Peng 2014)	Chengdu (a city in China)	1995–2012	Application rates of chemical fertilizers, pesticides and agricultural plastic films, density of slaughtered fattened hogs, and *per capita real GVA*	Non-linear regression analysis	The relationship between per capita real GVA and fertilizer input density reveals an N-shaped pattern. Inverted U-shaped curves were found between per capita real GVA and pesticide use intensity, agricultural film density, and density of slaughtered fattened hogs, respectively
(Shang et al. 2017)^b^	Heilongjiang province, China	1992–2014	Application rates of chemical fertilizers, pesticides and agricultural plastic film, density of livestock and poultry excrement, and *real per capita GVA*	ADF unit root test and regression analysis	Inverted U-shaped EKC was found between pesticide use and agricultural development. N-shaped EKCs were found between chemical fertilizer use, livestock and poultry excrement, and agricultural development, respectively
(Wang 2011)^b^	10 cities from Zhejiang province, China	2000–2008	Application rates of chemical fertilizers and pesticides, density of pigs and poultry, *real per capita GVA*, and rural population	Fixed effect model	The inverted U-shaped EKC was confirmed between agricultural pollution and agricultural growth for the study area. The per capita GVA at turning point was 6418.7 CNY. EKCs of all cities were before the turning point
(Xu et al. 2021)	30 Provinces in China	2006–2015	Nitrogen oxides emissions per capita from energy and nitrogen fertilizers, and GDP per capita	Panel unit root tests, panel cointegration test, and Dumitrescu–Hurlin causality tests	The inverted U-shaped EKC exists between economic growth and nitrogen oxides emissions in China. During the survey period, all provinces have reached their turning points
(Yao 2019)	31 provinces in China	2007–2016	N and P fertilizer pollution emission^a^, crop production value, and the proportion of crop production value in total agricultural output value	Fixed effects regression analysis	An inverted U-shaped EKC was confirmed between economic scale and non-point source pollution of chemical fertilizers
(Zhang and Hu 2020)	25 provinces in China	1995–2017	Fertilizer use intensity, per capita rural income, and urban–rural income gap	Dynamic panel-data model	An inverted U-shaped relationship exists between fertilizer use intensity and per capita rural income. However, the peak turning point is much higher than the actual per capita rural income of all provinces in China

^a^Fertilizer N and P surpluses were calculated differently in various studies. F. Li et al. (2016) estimated China’s N and P surpluses from synthetic fertilizers based on regional fertilizer input, nutrient uptake from crop products, and soil basic fertility. X. Zhang et al. (2015) established an N budget database for 113 countries. They quantified the N use efficiencies (NUE) for each country, taking into consideration the application rate of chemical and organic fertilizers, N fixation, atmospheric N deposition, and harvested N in yield Yao (2019) estimated the pollution emission of N and P fertilizers in China, by multiplying fertilizer application rates by fertilizer loss rates in the literature

^b^Original language in Chinese

^c^In this paper, the *gross output value of agriculture (GVA)* refers to the sum-up of the *gross output values of crop production (GVC)*, forestry, animal husbandry, and fishery

Since 2009, multiple studies included agrochemical consumption in the EKC context using time-series data. While some of those were conducted at a world scale covering various nations (see Table 1), many were specifically focused on China, either nationwide or region specific (see Table 2). For instance, Liu et al. (2009) investigated the relationship between chemical fertilizer consumption and per capita real gross output value of agriculture (GVA) of 31 provinces of China from 1949 to 2007, and concluded that 7 provinces had significant inverted U-shaped EKCs while 10 had N-shaped and linearly increasing curves. Li and Zhang (2009) tested the relationship between per capita GDP and chemical fertilizer use, pesticide use, and the density of livestock and poultry excrement, respectively. They found empirical support for the hypothesized inverted U-shaped EKC between China’s agricultural non-point source pollution and economic growth.

While the majority of the above-mentioned studies used the absolute application rates of fertilizers or pesticides as the environmental indicator, only a few adopted fertilizer pollution emission as an alternative (Zhang et al. 2015; Li et al. 2016; Celikkol Erbas and Guven Solakoglu 2017; Yao 2019). Zhang et al. (2015) established an N-budget database for 113 countries for the period 1961 to 2011, and used N surplus, i.e., the sum of N inputs minus N outputs (biologically fixed N and N deposition were also considered), as an environmental indicator in the EKC hypothesis. They investigated the EKC for each individual country using autoregressive distributed lag (ARDL) models and revealed a significant quadratic relationship between GDP per capita and N surplus. Similar to Zhang et al. (2015), Li et al. (2016) and Yao (2019) also adopted fertilizer surpluses instead of the application rates in their analysis. Both of the studies investigated the relationship between agricultural-related pollution and economic growth in China. In addition to fertilizer N surplus, they further included fertilizer P surplus and pesticide. Li et al. (2016) applied panel cointegration and panel-based dynamic OLS analysis, and Yao (2019) used fixed effects regression analysis. Both of their empirical findings revealed a long-run inverted U-shaped EKC in China between the environmental index and economic growth. Other than using fertilizer surpluses, Celikkol Erbas and Guven Solakoglu (2017) suggested an alternative path of quantifying the agricultural emissions. They estimated N_2_O emission from N fertilizers, and they investigated its relationship with economic development for 145 countries from 2002 to 2010. They revealed a short-run EKC relationship between agricultural emissions and income using vector error correction models (VECM).

## Research gaps

In the literature, we identified the following shortcomings or research gaps in the context of agrochemical consumption and the EKC hypothesis.

Firstly, the majority of studies used fertilizer application rates as the indicator of agricultural non-point source pollution. However, considering the inhomogeneity of nutrient use efficiencies among regions and over time, fertilizer nutrient surpluses are a better alternative. In addition, among the limited number of studies in which fertilizer surpluses were used as an indicator, only a few (e.g., Zhang et al. 2015) clearly stated how and from where the fertilizer surpluses data were derived. Secondly, in comparison to other EKC-related studies (e.g., EKC for greenhouse gas emissions), the majority of agrochemical-related EKC studies have rather short sampling periods (*T* < 25). This may reduce the robustness of their results considering that it is difficult to model nonstationarity in short time series. Nonetheless, this weakness might be unavoidable due to the lack of agrochemical data at a higher sampling frequency, and the fact that agrochemical overuse has only gained attention in recent decades (Yadav et al. 1997; Wu et al. 2018). Thirdly, various economic variables were used to indicate economic growth in the EKC context, especially in studies focused on China (see Table 2). The existing differences were mainly among real/nominal, GDP/GVA/GVC/farmers’ income, and per capita population/rural population. Therefore, the results should always be interpreted with caution and a comparison of EKC turning points across studies is difficult. Last but not least, while many EKC studies listed in Tables 1 and 2 account for the nonstationarity of the variables in the EKC regression (Gong and Tian 2010; Guo and Sun 2012; Shang et al. 2017), they still apply unit root and cointegration techniques that are not suitable for such cointegrated polynomial regressions (Wagner 2015). Particularly, they do not account for the fact that powers of an integrated process are in a deterministic relationship with the underlying integrated process which requires specific estimators (Wagner and Hong 2016).

## Methodology

### Theoretical framework and data

**Fig. 2:**
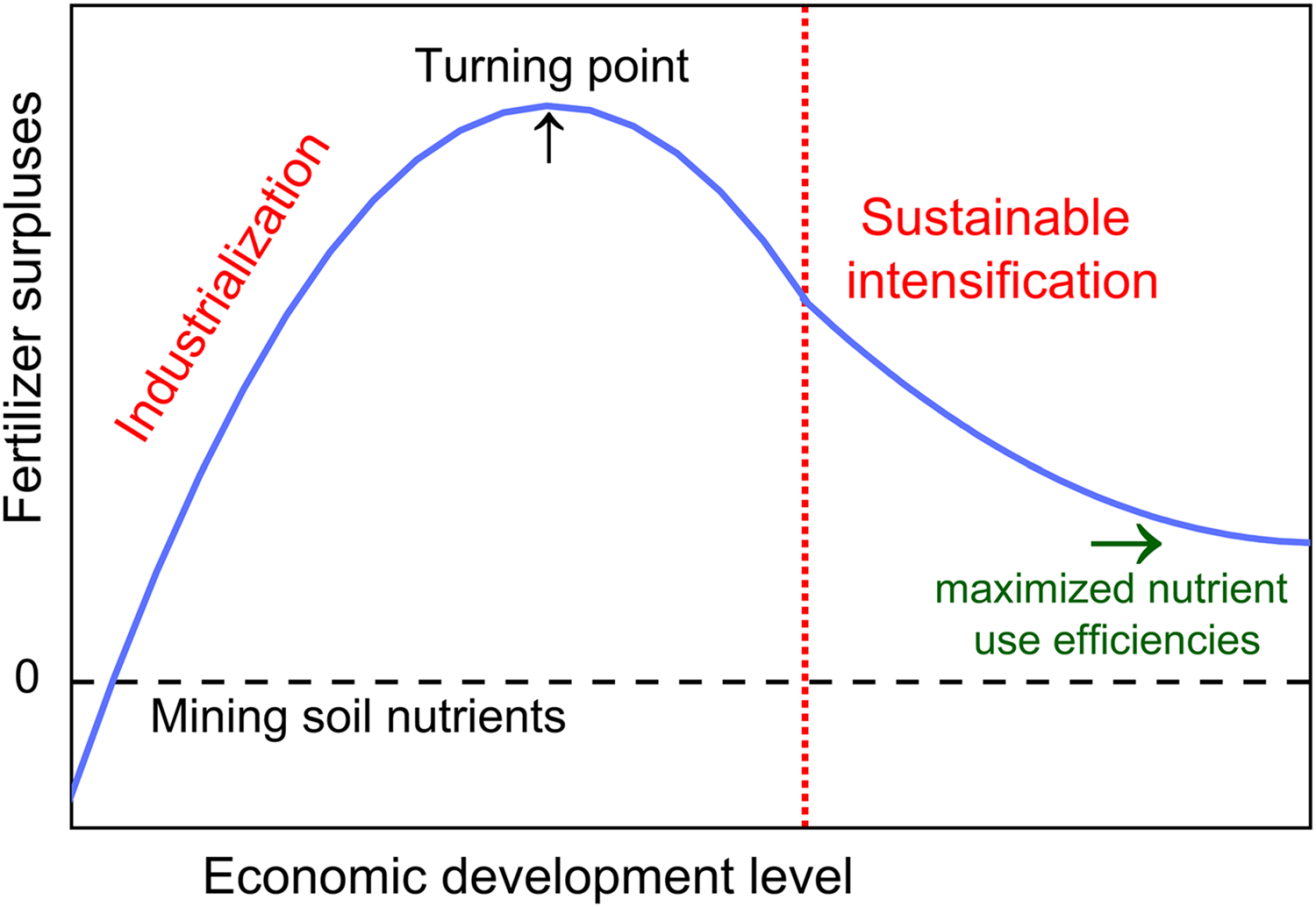
An idealized fertilizer surpluses-induced EKC. Modified from Zhang et al. (2015) and Murshed et al. (2021)

We hypothesize that the chemical fertilizer surpluses in China follow a similar pattern to the idealized EKC projection (Fig. 2). In the early stages of the industrialization, fertilizer surpluses increase rapidly with economic growth. This could be due to the increasing demand for food as the population grows, the expansion of nutrient-intensive cash crop production, and/or highly subsidized fertilizer prices. Then, as the economy further develops, the quest for, inter alia, environmental sustainability, and resource efficiency would emerge. This will slow down the increasing rate of fertilizer surpluses and eventually lead to the reduction. Later on, the fertilizer surpluses are expected to keep decreasing as a result of the sustainable intensification of agriculture throughout the post-industrialization period (Zhang et al. 2015; Murshed et al. 2021). It is important to understand the dynamic between economic development and fertilizer surpluses in the EKC context. This knowledge could help to evaluate the current fertilizer management and the resulting policy implications for China. Furthermore, understanding this relationship can provide a guideline towards a more sustainable agricultural production in the future.

The empirical analysis of the study employs balanced panel data covering 30 provinces of mainland China from 1988 to 2019 (Table 3). We selected real gross regional product (GRP) per capita as the indicator of economic development since it is a general indicator for economic progress at the regional level. The per capita GRP data were gathered from two sources: data from 1993 to 2019 were obtained directly from the National Statistical Bureau of China (NBS), and data from 1988 to 1992 were derived based on each year’s China Statistical Yearbook. All per capita GRPs were calculated at a constant price (1978 = 100).

**Table 3:** Description of the variables and data sources

Variable	Description	Data needed	Data source
Real GRP per capita (1000 CNY year^−1^)	Gross regional product per capita at a constant price (1978 = 100)	GDP of China^a^	NBS
		Indices of GDP (1978 = 100)	NBS
		GRP per capita	NBS (1993–2019), China statistic yearbooks (1988–1992)^b^
Fertilizer N and P surpluses (kg ha^−1^ year^−1^)	The difference between the sum of N and P inputs from chemical fertilizers, and the output from harvested crops	N and P from single-nutrient fertilizers	NBS
		N and P from compound fertilizers	NBS, China Agriculture Yearbook^c^
		Regional total sown area and regional orchard area^d^	NBS
		Regional NUE	Li et al. (2013a); Zhang et al. (2015)
		Regional PUE	Zhang et al. (2019)

^a^The GDP data of China were used to derived annual GDP deflators for further real GRP calculation, i.e., the GDP deflator of *t*-year is ${\mathrm{GDP deflator}}_{t}=\frac{{\mathrm{Nominal GDP}}_{\mathrm{t}}}{{\mathrm{GDP}}_{1978}\times {\mathrm{GDP Index}}_{\mathrm{t}}} \times 100$;

^b^Since GRP per capita data from 1988 to 1992 were not directly available, they were calculated by dividing the year’s GRP by the average value of the year’s year-end population and that of the previous year;

^c^N and P from compound fertilizers were estimated based on the N:P_2_O_5_:K_2_O ratio in different regions of China: 1:2.0:0.2 in the northeast region, 1:1.5:0.4 in the northcentral and northwest regions, and 1:1:0.8 in the middle and lower reaches of Yangtze River as well as the southwest and southeast regions (MOA 2010);

^d^According to NBS, the “total sown areas of farm crops” cover 10 categories of crops: grain, oil-bearing crops, cotton, hemp, sugar crops, tobacco, medicinal materials, vegetables, melons, and other farm crops (NBS 2020). The cultivation area of orchard fruits, e.g., apples, pears, and tropical fruits are not included. Therefore, we used the sum-up of total sown area and orchard area as the total cultivated land area. Note that tea plantations were not included in the calculation, considering their small fraction and lack of data in many regions

To address the environmental impacts due to the overuse of chemical fertilizers, we estimated the potential nutrient losses to the environment — N and P surpluses — from the applied chemical fertilizers. N and P surpluses (kg ha^−1^ year^−1^) are defined as a positive difference between N and P inputs and their outputs in crop production on an annual basis (Liu et al. 2010; Bouwman et al. 2013). By assuming that the differences among plant N and P use efficiencies (NUEs and PUEs) of various sources of N and P (e.g., chemical fertilizers, manures, etc.) are negligible, we quantified annual fertilizer N and P surpluses in agricultural soils of 30 provinces in China:

1 $${\mathrm{NP}}_{\mathrm{sur}(\mathrm{tj})}={N}_{\mathrm{fert}(\mathrm{tj}) }\bullet \left(1-{\mathrm{NUE}}_{\mathrm{tj}}\right)+{P}_{\mathrm{fert}\left(\mathrm{tj}\right)}\bullet \left(1-{\mathrm{PUE}}_{\mathrm{tj}}\right),$$

where NP_sur(tj)_ (kg ha^−1^) is the sum of N and P surpluses from chemical fertilizers of province j in year *t*. *N*_fert_ and *P*_fert_ (kg ha^−1^) refer to the effective components of fertilizer N and P from single-nutrient and compound fertilizers. NUE_tj_ and PUE_tj_ are the regional N and P use efficiency in year t. The regional NUEs of China used in this study were derived from Zhang et al. (2015) and S. Li et al. (2013a), and the regional PUEs were estimated by Zhang et al. (2019). Fertilizer input data as well as regional land use data were attained from NBS. Note that a surplus of potash fertilizer was not considered in the study, given that potash in agricultural soils in China has been deficient in most of the regions (He et al. 2015). For simplicity, the Chongqing region was included in Sichuan province.

For a more detailed description concerning the calculation of the regional N and P surpluses in China, see Yu et al. (2020).

All variables were transformed into their natural logarithm for further analysis.

### Unit root tests, cointegrating polynomial regressions, and panel analysis

Considering the criticism regarding the use of nonstationary data in some classic EKC-related studies (Müller-Fürstenberger and Wagner 2007; Wagner 2008, 2015), we performed unit root tests for our data to test the variables for their order of integration. We applied three types of unit root tests: the Phillips–Perron (PP) and augmented Dickey-Fuller (ADF) tests, testing the null hypothesis of a unit root; and the Kwiatkowski–Phillips–Schmidt–Shin (KPSS) test, testing the null hypothesis of mean (trend) stationarity. All tests were conducted with a constant and trend specification.

After determining the order of integration, we specified the EKC regression for each province. Following Wagner (2015), the classical EKC regression is a cointegrating polynomial regression, and its coefficients and standard errors need to be estimated with a specific methodology. For this purpose, we implemented the FM-OLS approach proposed by Wagner and Hong (2016) based on the following equation:

2 $${\mathrm{NPsur}}_{\mathrm{it}}={\beta }_{0}+{\beta }_{1}{\mathrm{GRP}}_{\mathrm{it}}+{\beta }_{2}{\mathrm{GRP}}_{\mathrm{it}}^{2}+{e}_{\mathrm{it}},$$

where ${\mathrm{GRP}}_{\mathrm{it}}$ is assumed to be integrated of order one and ${e}_{\mathrm{it}}$ is a stationary error term. The variables were log-transformed before they entered the regression. We then tested for cointegration using either the ${P}_{\mathrm{u}}$ statistic (null hypothesis of no polynomial cointegration) or the KPSS-type statistic (null hypothesis of polynomial cointegration) suggested by Wagner (2015) and Wagner and Hong (2016). Should the null hypothesis of the ${P}_{\mathrm{u}}$ test be rejected, or the null hypothesis of the KPSS-type test not be rejected (ideally both test decisions should align), we conclude that $\mathrm{GRP}$ and $NPsur$ hold a stable non-linear long-run relationship over the sampling period. Otherwise, the error terms would be deemed nonstationary and Eq. 2 would represent a spurious regression purely driven by the variables trending in the same direction. After cointegration testing, we need to further classify the estimated EKCs according to their shape. For this purpose, we need to conduct valid inference on the coefficients of the EKC regression which is guaranteed (at least asymptotically) by using the FM-OLS estimator. Conceptually, the FM-OLS estimator uses a two-part transformation to dynamically orthogonalize the variables and removes an additive bias term to allow for standard asymptotic inference. For these non-parametric corrections, long-run variances need to be estimated. Following Grabarczyk et al. (2018), we estimate all long-run variances based on the Bartlett kernel and the data-dependent bandwidth rule of Newey and West (1994). Specifically, we tested whether the coefficient ${\beta }_{2}$ is significantly different from zero to distinguish linear from quadratic curves. Only if the null hypothesis ${\beta }_{2}=0$ is rejected, the conditions ${\beta }_{1}>0$ and ${\beta }_{2}<0$ hold, we can classify the estimated curve as being consistent with the inverted U-shape suggested by the EKC hypothesis. We can then use our coefficient estimates to calculate the turning points,$\mathrm{exp}(\frac{{-\beta }_{1}}{2{\beta }_{2}})$ for each EKC relationship.

Since we are limited to a short sampling period but have a panel structure with 30 provinces, we also estimated several panel cointegration models according to Wagner and Reichold (2018) to benefit from the available cross-sectional dimension. To do so, we computed the cross-sectional average over the FM-OLS coefficients obtained from the individual EKC regressions. Moreover, they provide the necessary tools to conduct inference. These results can then be compared to EKC regressions for yearly country-level data employed in the literature (Gong and Tian 2010; Guo and Sun 2012; Li et al. 2016). In addition, we also computed the group-mean estimates over the six regions in China, i.e., Northeast, Northcentral, Middle and lower reaches of Yangtze River, Southeast, Northwest, and Southwest zones. This helped to gain more insights into the regional dispersion of the results and to investigate the spatial pattern.

For the robustness check of the $\mathrm{GRP}$−$\mathrm{NPsur}$ nexus from Eq. 2, we further introduced a control variable into the model. Similar to Celikkol Erbas and Guven Solakoglu (2017), we included the share of crop production sector to GRP ($\mathrm{CropGRP}$) to incorporate the level of agricultural activities in regional economies. The modified model can be specified as:

3 $${\mathrm{NPsur}}_{\mathrm{it}}={\beta }_{0}+{\beta }_{1}{\mathrm{GRP}}_{\mathrm{it}}+{\beta }_{2}{\mathrm{GRP}}_{\mathrm{it}}^{2}+{\gamma {\mathrm{CropGRP}}_{\mathrm{it}}+e}_{\mathrm{it}},$$

We derived $\mathrm{CropGRP}$ for 30 provinces of China from 1988 to 2019. After determining the order of integration for $\mathrm{CropGRP}$, we performed the earlier described FM-OLS approach for the coefficient estimations. Signs of the coefficients and their significant levels from Eq. 3 will be later compared with the main results from Eq. 2 for a robustness check.

## Results

### Unit root tests

We conducted three types of unit root tests for each regional variable. The test specification either included a constant or a constant with linear trend term. The results are presented in Table 7 in the Appendix. The PP tests showed that the NPsur variables of 14 provinces, and the GRP variables of 29 provinces were determined to have a unit root, but both variables turned stationary after first differencing for the large majority of the provinces. The ADF test results are similar to those of the PP test for the variables in levels. However, only less than half of the provinces’ NPsur and GRP are determined to be integrated of order one, according to the ADF results. In contrast to the ADF and PP test, the KPSS test has the null hypothesis that the stochastic process generating the respective time series is mean-stationary or trend-stationary depending on the exact specification of the test regression. Our results showed that the null hypothesis cannot be rejected for both variables in their level form if we include only a constant term. However, the results were more ambiguous when we included an additional linear trend term. Here, the results agreed with the PP test results for NPsur, but we only determined a minority of provincial GRP variables to be nonstationary. Moreover, only a couple of GRP variables were found to be stationary after first differencing. Although some provinces had variables that were stationary according to the ADF, PP, and KPSS test, we argue that the variables were integrated of order one, which is the conservative choice considering that the polynomial EKC regression can be estimated straightforwardly for stationary variables as well. The main reason for our argument is that, due to the data availability, our sample size was limited to *T* = 30, which is very small in the context of unit root testing. Unit root tests are known to suffer from size distortions and low power when the sample size is too small. Particularly, the KPSS test has unfavorable small sample properties (Caner and Kilian 2001). Secondly, there was no overlap of nonstationarity results among the three tests; in other words, both variables of all the provinces tested nonstationary in at least one test.

### Estimating the relationship between chemical fertilizer surpluses and economic growth in China

Table 4 presents the results of both polynomial cointegration tests and parameter estimates for each province using Eq. 2. The KPSS-type cointegration tests for each province showed that the residuals of the EKC regressions follow a stationary trajectory. This means that a significant cointegrated relationship between fertilizer surpluses and per capita GRP was maintained for all provinces. The corresponding test with the opposing null hypothesis of no cointegration $({P}_{u}$ statistic) again suffers from low power in small samples. In this case, we can only reject the null hypothesis for two provinces at the 5% level. However, we closely studied the residuals for those provinces where the results of the two cointegration tests disagreed and found that their trajectory much more resembled that of a typical stationary process. Therefore, we assume that the variables are cointegrated and that the opposing test results were driven by the small sample size which makes it difficult to distinguish between stationary and nonstationary processes. Moreover, these ambiguous results are not surprising if we take into account that the results for the preceding unit root tests were ambiguous as well.

**Table 4:** Results of cointegration tests and CPR coefficient estimates

	Cointegration tests		FM-OLS estimates		
Province	${P}_{u}$ statistic	KPSS statistic	*β*_0_	*β*_1_	*β*_2_
Liaoning	10.731	0.065**	4.46***	0.52***	− 0.12***
Jilin	16.169	0.058**	4.40***	0.25***	− 0.05
Heilongjiang	9.391	0.073**	3.63***	0.49***	− 0.14**
Beijing	9.507	0.07**	4.41***	0.64**	− 0.09
Tianjin	10.158	0.102**	3.10***	1.84***	− 0.34***
Hebei	13.579	0.061**	4.71***	0.58***	− 0.16***
Shanxi	9.063	0.053**	4.60***	0.49***	− 0.14***
Shandong	10.869	0.088**	4.89***	0.6***	− 0.18***
Henan	7.844	0.037**	5.00***	0.51***	− 0.13***
Shanghai^a^	17.939	0.087**	4.86***	0.65	− 0.2**
Jiangsu	4.215	0.063**	5.02***	0.63***	− 0.18***
Zhejiang	15.322	0.104**	4.79***	0.23**	− 0.01
Anhui	13.45	0.082**	4.96***	0.38 ***	− 0.15***
Jiangxi	16.716	0.065**	4.58***	0.32***	− 0.15***
Hubei	9.406	0.058**	5.02***	0.72***	− 0.26***
Hunan	13.944	0.069**	4.67***	0.34***	− 0.12***
Fujian	21.169*	0.089**	5.04***	0.22***	0.03
Guangdong^b^	18.605	0.083**	4.99***	0.1	0.05
Guangxi	16.175	0.096**	4.71***	0.34***	− 0.04*
Hainan	9.183	0.141**	4.63***	0.79***	− 0.12
Sichuan	20.72*	0.061**	4.78***	0.34***	− 0.12***
Guizhou	15.254	0.059**	4.56***	0.2***	− 0.16***
Yunnan	17.778	0.097**	4.68***	0.48***	− 0.13***
Tibet	28.43**	0.059**	4.36***	0.77***	− 0.26***
Inner Mongolia	9.873	0.053**	4.04***	0.62***	− 0.1***
Shaanxi	10.485	0.063**	4.80***	0.6***	− 0.14***
Gansu	18.748	0.044**	4.56***	0.55***	− 0.25***
Qinghai	23.509**	0.064**	4.29***	0.5***	− 0.18***
Ningxia	17.522	0.062**	4.68***	0.51***	− 0.18***
Xinjiang	12.092	0.057**	4.33***	0.85***	− 0.21***

Triple, double, and single denote statistical significance at 1%, 5%, and 10%, respectively

^a^^,^^b^Since the linear terms of Shanghai and Guangdong were insignificant in the quadratic model specification, we conducted *F*-tests of the joint hypothesis that both coefficients are zero for the two provinces. The null hypothesis was rejected for both provinces (*p* < 0.001), so we re-estimated the linear model specification. The *t*-statistics showed that the linear term was significant for both provinces and the parameter estimates were Guangdong: NPsur = 4.921 + 0.236GRP, and Shanghai: NPsur = 5.687–0.200GRP

The FM-OLS estimates in Table 4 indicate that 22 provinces out of 30 exhibit an inverted U-shaped EKC between GRP and NPsur (*p* < 0.05), 7 provinces showed positive linear relationship whereas 1 province exhibit a negative linear relationship between GRP and NPsur. These results are largely in line with those from the robustness check using Eq. 3 (see Tables 8 and 9 in the Appendix), where 23 provinces (the same 22 provinces plus Beijing) showed a significant inversed U-shaped EKC. Results of the linear specification of Eq. 3 also showed similarities to those using Eq. 2, despite that some linear coefficients of Eq. 3 are insignificant. This results further established the robustness of our results using Eq. 2.

Table 5 reports the group-mean panel FM-OLS results for China and the six regions. The panel results for five out of six regions imply an inverted U-shaped EKC.

**Table 5:** Panel results based on the group-mean FM-OLS

	Provinces included	*β*_0_	*β*_1_	*β*_2_	Turning point (in CYN per capita, 1978 = 100)
Northeast	Jilin, Liaoning, Heilongjiang	4.16***	0.42***	− 0.11***	7022
Northcentral	Beijing, Tianjin, Hebei, Shanxi, Shandong, Henan	4.45***	0.78***	− 0.17***	9726
Northwest	Shaanxi, Gansu, Qinghai, Ningxia, Xinjiang, Inner Mongolia	4.45***	0.61***	− 0.18***	5588
Middle and lower reaches of Yangtze River	Shanghai, Jiangsu, Anhui, Jiangxi, Hubei, Hunan	4.84***	0.47***	− 0.15***	4697
Southeast	Zhejiang, Fujian, Guangdong, Guangxi, Hainan	4.84***	0.36***	− 0.02	−
Southwest	Sichuan, Guizhou, Yunnan, Tibet	4.60***	0.45***	− 0.17***	3749
China		4.59***	0.54***	− 0.14***	6705

Providing the existence of a significant cointegration between fertilizer surpluses and the economic growth of all the provinces, we categorized the provinces into four groups. Similar to the approach of Zhang et al. (2015), we grouped the provinces based on the significance and the sign of the coefficients of the linear and quadratic terms. Provinces with a significant inverse U-shaped EKC (*p* < 0.05) were defined as Group 1 — *inverted U-shaped curves*. Provinces that exhibit significant linear relationships between GRP and NPsur were categorized as *Group 2 — linearly increase* or *Group 3 — linearly decrease,* depending on the sign of the linear coefficient*.* If none of the quadratic or linear terms were significant, we conducted an *F*-test of the joint hypothesis that both terms are insignificant. If this null hypothesis was not rejected, that province was relegated to *Group 4 — insignificant.*

To indicate whether the fertilizer surpluses of a province had passed the peak and started to decline as the economy grew, we further characterized Group 1 — inverted U-shaped curves into three subgroups. We compared those provinces’ recent 3-year (2017–2019) and 5-year (2015–2019) averages of fertilizer surpluses and per capita GRP with their corresponding estimated EKC peak positions. A province was considered to *have passed the peak (Group 1a)* if the following two conditions were met: (1) both of its 3-year and 5-year averages of fertilizer surpluses were lower than the lower bound of the 95% confidence interval for the estimated fertilizer surpluses peak, and (2) both of its 3-year and 5-year per capita GRP averages were higher than the per capita GRP at the peak $(\frac{{-\beta }_{1}}{2{\beta }_{2}})$. Similarly, a province was considered to be *at the peak (Group 1b)*, if *only* its 3-year averages of fertilizer surpluses and per capita GRP fulfilled the previously described criteria. Lastly, a province was considered to be *before the peak (Group 1c)*, if it did not fall into the former two subgroups.

**Fig. 3:**
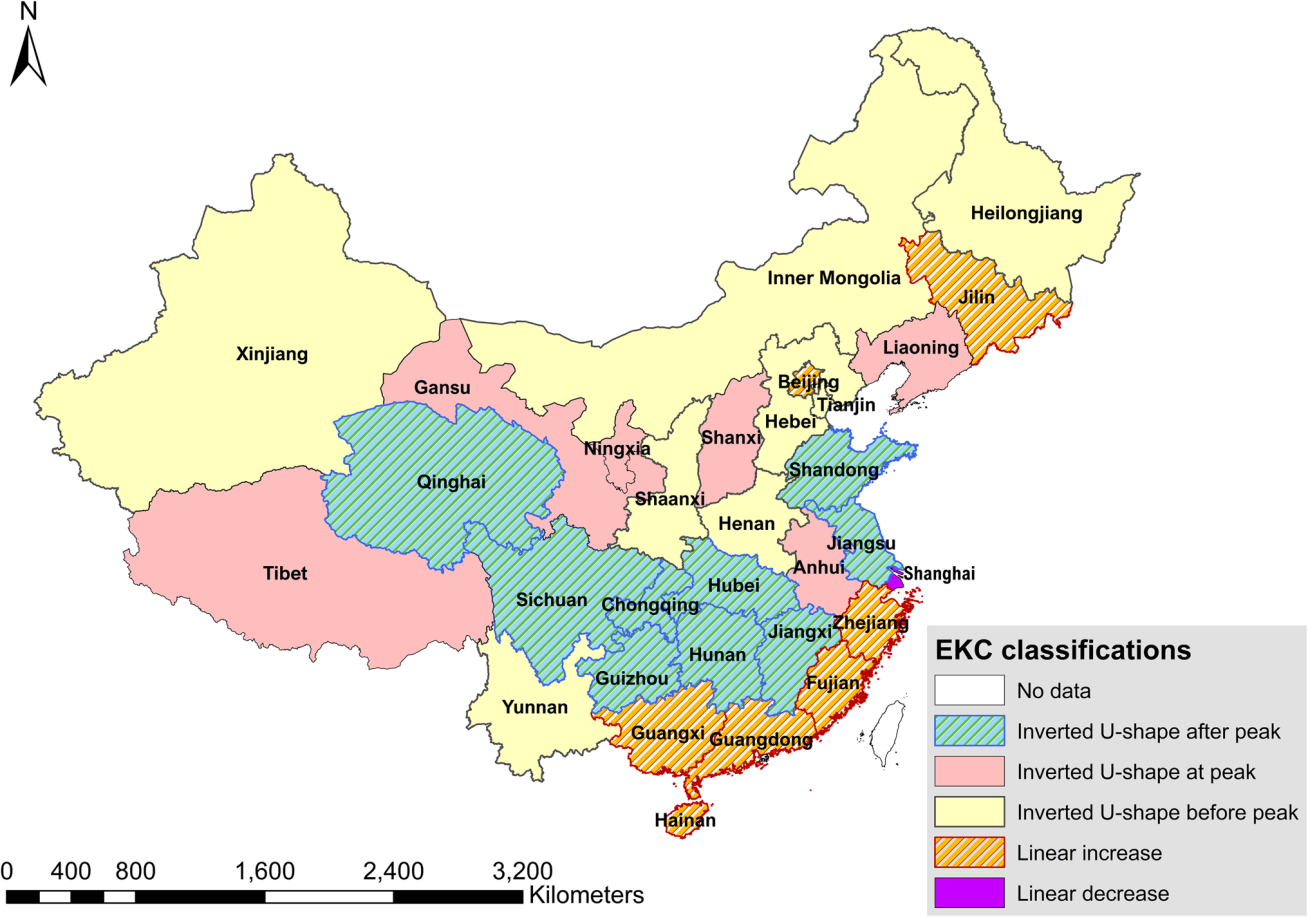
The distribution of different shapes of EKCs in China. Source of data: own calculation

**Fig. 4:**
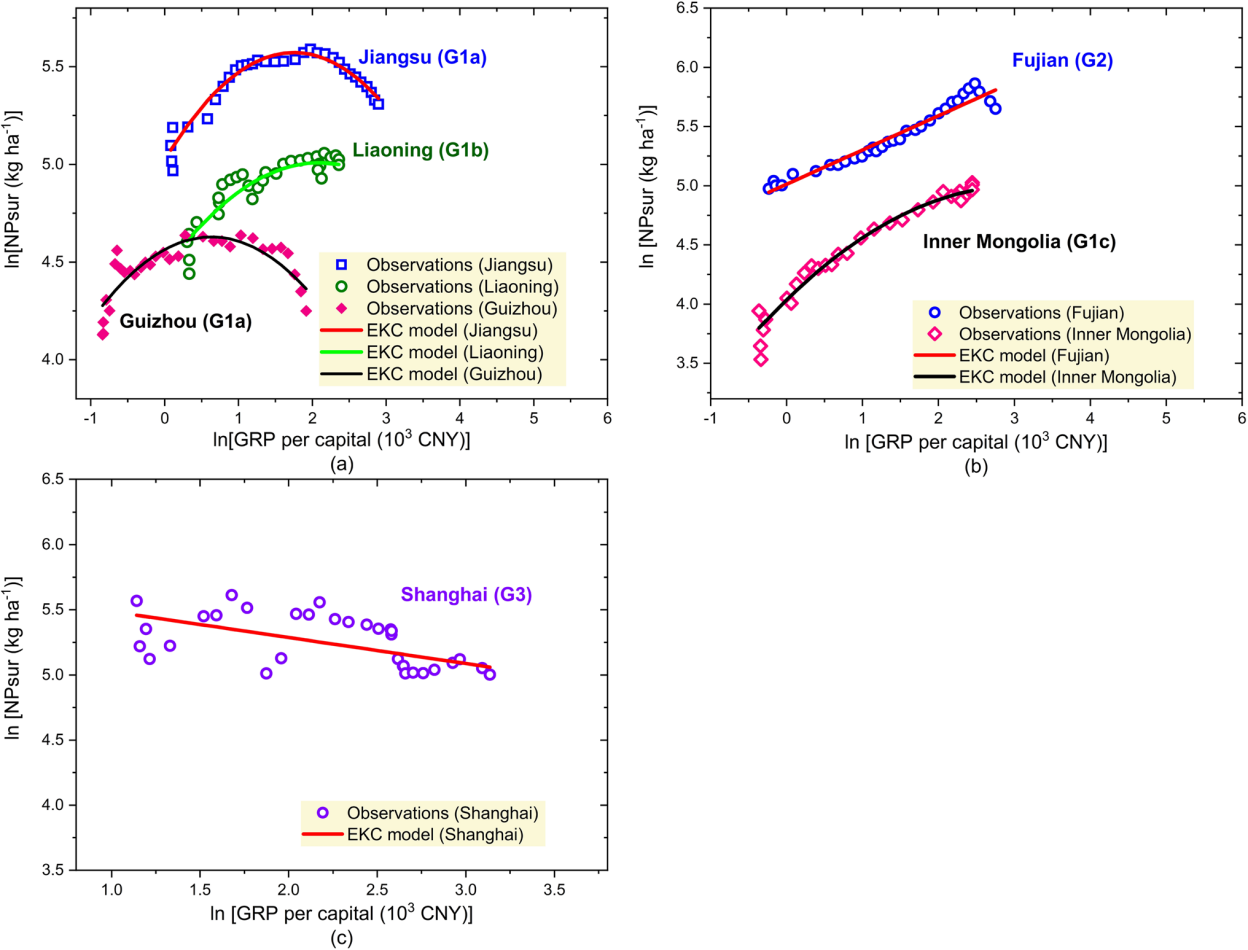
Examples of the relationships between economic growth and fertilizer surpluses. **a** Comparison between Jiangsu and Guizhou in *Group 1a — inverted U-shaped curves after peak*, and Liaoning in *Group 1b — inverted U-shaped curve at peak.* **b** Comparison between Inner Mongolia in *Group 1c — inverted U-shaped curve before peak*, and Fujian in *Group 2 — linear increase*. **c** Shanghai in *Group 3 — linear decrease*

Figure 3 illustrates the distribution of various response types of EKCs over China. Among the 22 provinces with significant inverted U-shaped EKCs, 8 provinces were considered to have passed the peak (Group 1a: Shandong, Jiangsu, Jiangxi, Hubei, Hunan, Qinghai, Sichuan, and Guizhou), indicating fertilizer surpluses of these regions had been decreasing as the economy developed. Six provinces were considered to be at the peak with the tendency of levelling off (Group 1b: Liaoning, Shanxi, Anhui, Gansu, Ningxia, and Tibet). Examples of provinces in Groups 1a and 1b are shown in Fig. 4 a. The provinces belonging to Groups 1a and 1b have around half of the cultivated land in China, covering the region of the middle and lower reaches of Yangtze River, a large fraction of southwest China, and some parts of the northcentral, northeast, and northwest China. Besides Groups 1a and 1b, eight provinces fell into Group 1c — inverted U-shape before peak, suggesting that the fertilizer surpluses of those provinces will still increase as the economy grows but with diminishing increasing rates. With the exception of Yunnan province, the majority of the Group 1c provinces are located in northern China, including Heilongjiang, Henan, Tianjin, Hebei, Inner Mongolia, Shaanxi, and Xinjiang. In 2019, those 22 provinces with inverted U-shaped EKCs covered 86.3% of the cultivated land in China and were responsible for 82.5% of the total chemical fertilizer consumption. Their total GVC accounted for 82% of the domestic GVC (NBS 2020). These data provide support for a classic EKC relationship between agricultural pollution and economic development. Considering that most of the regions in China have already achieved negative annual growth rates of fertilizer consumption as a response to the “Zero growth plan for fertilizers by 2020” (Jin et al. 2019; Yu et al. 2020), those results further support the hypothesis that chemical fertilizer management has improved in China since 2015. In contrast to those findings, one province — Shanghai — exhibited a decreasing linear relationship between per capita GRP and fertilizer surpluses (see Fig. 4c).

Nevertheless, in addition to the inverted U-shaped and linear decreasing curves, seven provinces still showed a positive linear relationship between fertilizer surpluses and economic development (Group 2). These provinces include Guangdong, Zhejiang, Fujian, Guangxi, and Hainan in Southeast China as well as Jilin and Beijing in the north. In contrast to the provinces in Group 1c, those in Group 2 tended to have constant increases in fertilizer surpluses as the per capita GRP grew (see Fig. 4b). The current observations suggest that an occurrence of EKC turning points for these seven provinces is unlikely in the near future.

## Discussion

Although the existence of an inverted U-shaped relationship between economic and environmental indicators has been empirically supported in various studies, the potential factors influencing the shapes and turning points of EKCs are controversially discussed in the literature. Many have suggested that the potential causes are complex and may be affected conjointly by, inter alia, environmental and socio-economic conditions, cultures, technologies, international trades, and policies (Dinda 2004; Kaika and Zervas 2013; Sarkodie 2018). This makes it difficult to evaluate horizontal comparisons of EKC results among different nations or regions.

Over the last two decades, there has also been a sizeable amount of literature criticizing the EKC hypothesis. One common criticism was that the shapes and turning points of the EKCs vary notably depending on the chosen indicators and the scale of the studies, making it difficult to find a specific value as a good predictor of the EKC turning points among nations (Dinda 2004; Zhang et al. 2015). Another criticism concerned the incomparability of EKCs between developed and developing countries. For instance, Stern (2004) argued that the EKCs estimated for developing countries are more likely to show an inverted U-shape than those for developed countries because the former could adopt the latter’s technological innovations to reduce pollution with a short time lag. Furthermore, Nahman and Antrobus (2005) suggested that the EKC hypothesis is a “historical artifact” resulting from the relocation of pollution-intensive industries from developed to developing countries.

Nevertheless, the objective of the present study was not to investigate the validity of the EKC theory. Our intention was to visualize and discuss the interconnections among chemical fertilizer consumption, socio-economic development, cropping structures, and policy implications using the existing EKC framework. In addition, our focus was specifically on regional China, which narrowed down the potential factors that may confound our empirical results.

### Socio-economic development, policy interventions, and the turning points on the EKC

**Fig. 5:**
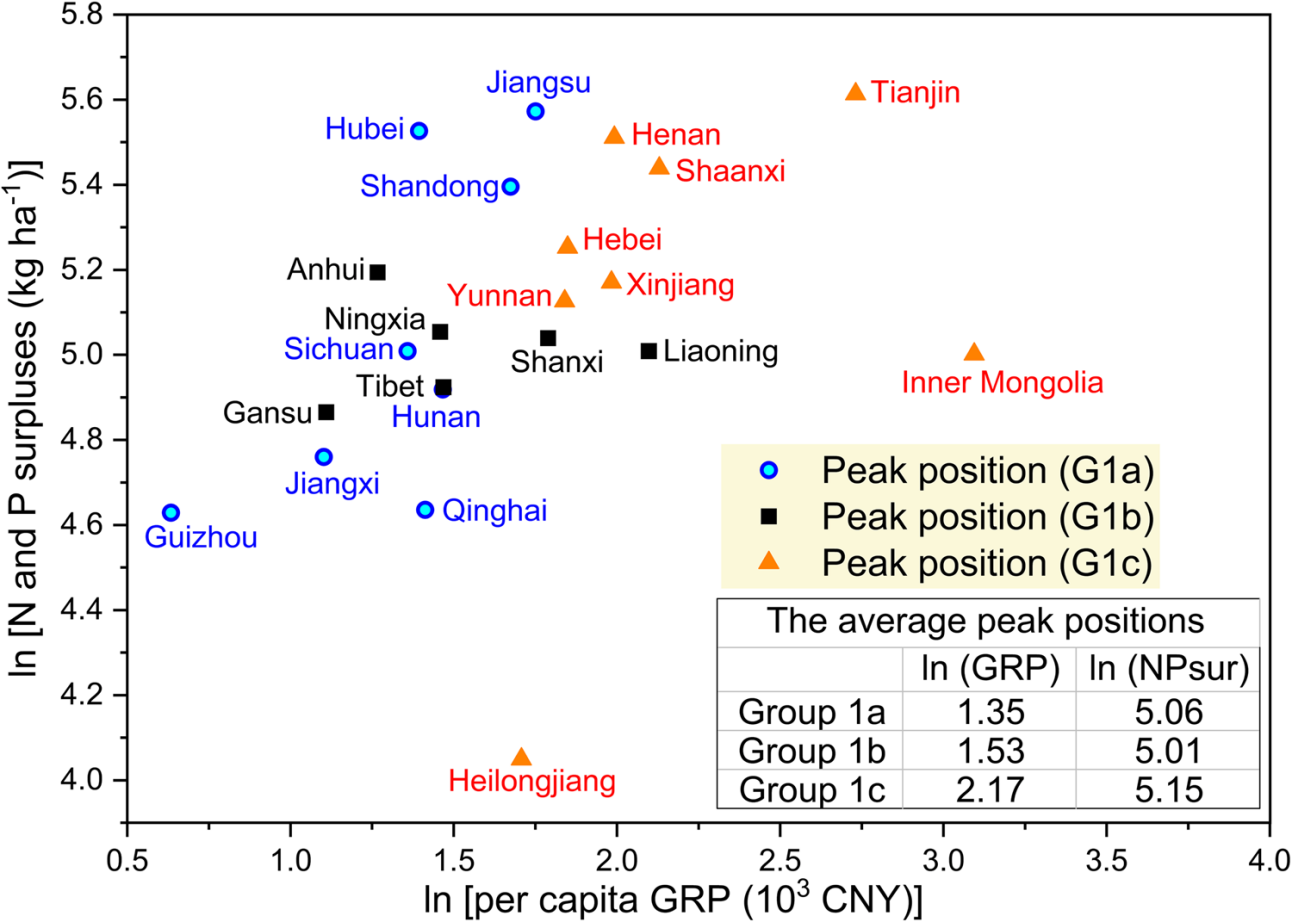
The estimated peak positions (turning points) of the provinces with inverted U-shaped EKCs between fertilizer surpluses and economic growth

Our results suggest that a total of 22 provinces in China out of 30 had a significant inverted U-shaped relationship between fertilizer surpluses and economic development. Yet, the modeled peak positions, i.e., turning points on the EKCs, appeared to have large variances among and within the groups (see Fig. 5). Generally, provinces belonging to Group 1a reached the turning point at lower levels of per capita GRP (1978 = 100) in comparison to the provinces belonging to Groups 1b and 1c. In addition, the provinces in Group 1c have the highest average turning point both in terms of per capita GRP and fertilizer surpluses. On average, the fertilizer surpluses of provinces in Group 1a started to decrease when the real GRP reached CNY 4044 per capita, and the corresponding value was CNY 9820 for provinces in Group 1c. Considering that large areas in provinces of Group 1c are regarded as socio-economically less-developed regions of China — especially in Heilongjiang, Henan, Shaanxi, Xinjiang, and Yunnan (Gu et al. 2011; Tian et al. 2020), the high per capita GRP at the turning point added uncertainties in their future EKC projections.

The variations in turning points also appeared on the regional level (see Table 5). For example, since the large majority of provinces in the region of Lower and middle reaches of Yangtze River have reached their turning points, this region has a rather low level of group-mean GRP at the turning point (CNY 4697 per capita). On the contrary, the estimated turning point of the Northcentral region is CNY 9726 per capita, as three out of five provinces there have not reached their respective turning points yet. Our group-mean panel results showed that the overall turning point for China is CNY 6705 per capita real GDP, which was reached in circa 2012. This result is basically in line with the reported turning points for agrochemicals in China from other studies (Gong and Tian 2010; Wang 2011; Hong 2013; Li et al. 2016). Although it appears difficult to compare the exact peak values due to various indicators selected, the estimated time points of the peak position are similar. For example, Li et al. (2016) concluded that China had reached its turning point of nitrogen indicator in 2009 whereas the turning point of phosphate indicator was still far ahead. Gong and Tian (2010) argued that China’s turning point of fertilizer consumption had been reached in 2008, whereas Li and Zhang (2009) noted that in 2005 the fertilizer-induced EKC was still in the increasing phase. Some regional studies indicated that Chongqing had reached its turning point of fertilizer input in 2009 (Hong 2013), and Jiangsu was approaching the turning point in 2010 (Guo and Sun 2012).

In addition, we also found that provinces that still exhibit positive relationships between fertilizer surpluses and economic growth, i.e., the provinces in G1c and G2, have generally large proportions of gross output value of crop production over the total gross regional product. For example, the three provinces with the highest ratio of GVC to GRP in 2019 were Heilongjiang (0.28) and Xinjiang (0.19) from Group 1c, and Hainan (0.15) from Group 2, in comparison to the national average 0.07 (NBS 2020). The reliance of those regions on crop production also makes it more challenging to reduce fertilizer consumption.

**Fig. 6:**
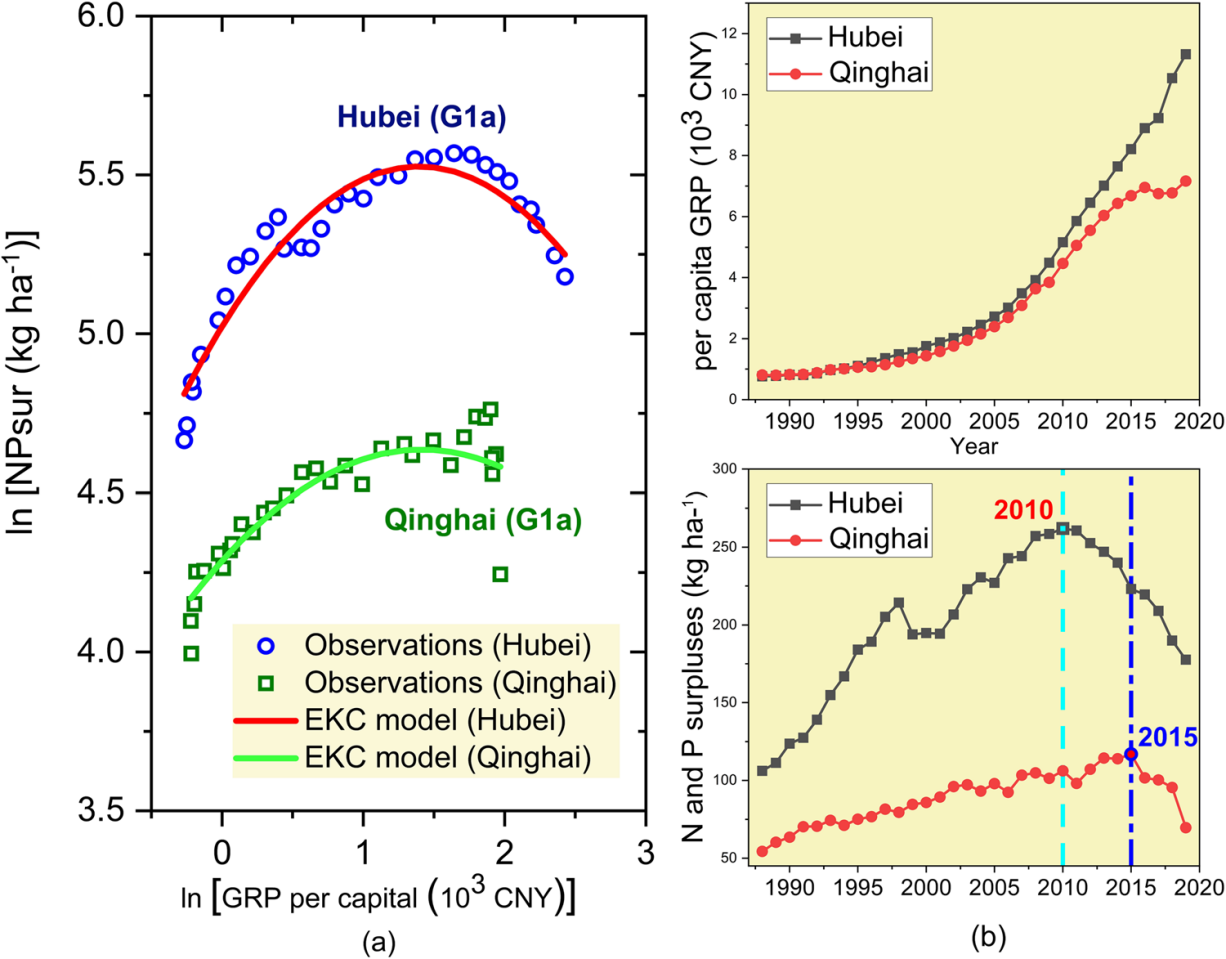
**a** EKCs of Hubei and Qinghai and **b** their corresponding records of per capita real GRP and fertilizer surpluses. Source of data: NBS and own calculation

Besides the differences of turning points among the subgroups, there are also large variations within each group. For example, both Hubei (G1a) and Qinghai (G1a) reached the turning point when the real GRP was approx. CNY 4000 per capita, but the peak fertilizer surpluses of Hubei was 251 kg ha^−1^, 2.4 times of that of Qinghai (103 kg ha^−1^). Figure 6 illustrates the EKCs of Hubei and Qinghai as well as their corresponding records of per capita real GRP and fertilizer surpluses from 1988 to 2019. While both provinces’ per capita real GRP increased steadily at different paces, their fertilizer surpluses started to decline at different time points. The fertilizer surpluses of Hubei reached their peak in 2010. During 2011 to 2014, the average annual growth rate of fertilizer surpluses was − 2%, which increased to − 5% during 2015 to 2019. On the contrary, the fertilizer surpluses of Qinghai only started to decrease in 2016 — 1 year after the implementation of the “Zero growth plan for fertilizers by 2020.” During 2016 to 2019, Qinghai enjoyed a sharp decline in fertilizer surpluses with a mean annual reduction rate of 12%. It can be argued that, without policy interventions, the fertilizer surpluses of Qinghai might have still increased, or reduced at a lower speed, which would have possibly shifted its EKC turning point to a higher level of GRP. On the other hand, the policies may have only accelerated the fertilizer use reduction of Hubei, without making any great impact on its turning point.

Sharp drops in fertilizer surpluses after 2015 were also found in Jiangxi and Guizhou of Group 1a, all provinces of Group 1b, and most of the provinces in Group 1c. The gradual implementation of the “Zero growth plan for fertilizers by 2020” since 2015 seemed to have played a role in shifting the potential EKC turning points to lower positions nationwide. Yet, its influences may vary depending on the regional governmental executive abilities and cropping structures. These results emphasize that such policies can have positive effects on fertilizer reduction in China and on the EKC shapes. However, more research needs to be conducted to estimate the causal effects of this policy.

One special case in our results is the case of Shanghai, where a negative linear relationship between per capita GRP and fertilizer surpluses was found. To the best of the authors’ knowledge, such a relationship was not indicated in other EKC-related studies. One possible explanation would be that Shanghai, as one of the most economically developed regions in China, had its potential turning point far before our sampling period. Therefore, only the “tail” of the inverted U-shaped curve was captured. Nevertheless, due to data restrictions we could not further investigate this issue.

### The EKCs and crop mix

Another factor that may be closely associated with the response types of the EKC is the crop mix (Zhang et al. 2015; Sarkodie 2018). Since the 1990s, the monoculture of cash crops in China quickly expanded due to its profitability and the improved agricultural infrastructures such as drip irrigation (Wang et al. 2004; Su et al. 2016). The expansion was especially notable in tropical and subtropical China as well as in northwest China. The former had massive tracts of arable land transformed to plantations for commercial fruits, palm oil, and rubber (Su et al. 2011; Yang et al. 2016), and the latter is the most dominate cotton-growing region in China (Feng et al. 2017). During the time span from 2017 to 2019, the southeast and southwest regions had the highest cash-crop ratio in China (0.60 and 0.44), followed by the northwest region 0.42 (see Table 6).

**Table 6:** Average cash-crop ratios and fertilizer surpluses of the six zones of China. According to NBS, farm crops can be divided into grain crops and cash crops. Grain crops include cereal grains, pulses, and tubers while cash crops include oil crops, fruits and vegetables, cotton and hemp, sugar crops, tabaco, and medicinal herbs. The cash-crop ratio here is calculated as $$1-[sown area of grain crops/\left(total sown areas of farm crops+orchard area\right)]$$ of the corresponding regional scale. Calculations were based on a 3-year average between 2017 and 2019. Source of data: NBS and own calculation

Zone	Provinces included	Cash-crop ratio	Fertilizer N and P surpluses (kg ha^−1^)
Northeast	Jilin, Liaoning, Heilongjiang	0.08	81.85
Northcentral	Beijing, Tianjin, Hebei, Shanxi, Shandong, Henan	0.27	204.58
Northwest	Shaanxi, Gansu, Qinghai, Ningxia, Xinjiang, Inner Mongolia	0.42	158.73
Middle and lower reaches of Yangtze River	Shanghai, Jiangsu, Anhui, Jiangxi, Hubei, Hunan	0.34	161.96
Southwest	Sichuan, Guizhou, Yunnan, Tibet	0.44	127.67
Southeast	Zhejiang, Fujian, Guangdong, Guangxi, Hainan	0.60	212.85
China average		**0.34**	**159.28**

**Fig. 7:**
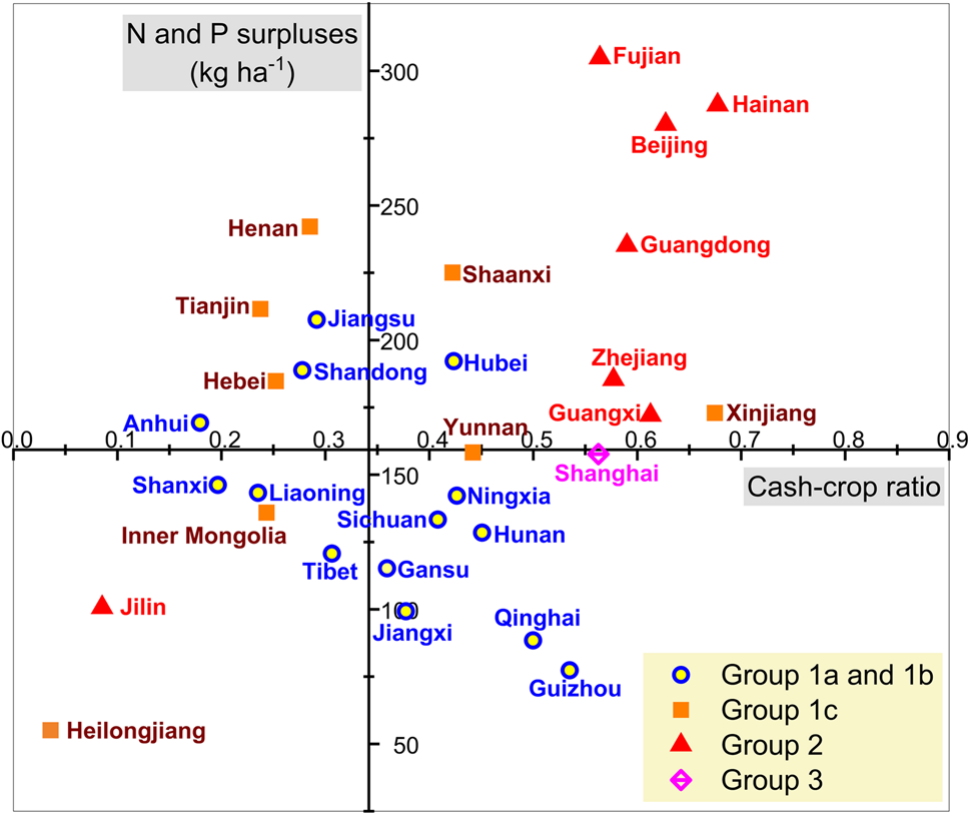
Scatter plot of all provinces’ cash-crop ratios (*x*-axis) versus fertilizer N and P surpluses (*y*-axis). The origin is located at the average values of the cash-crop ratio and fertilizer N and P surpluses of China, where *x* = 0.34 and *y* = 159.28. All of the data points were calculated based on a three-year average between 2017 and 2019. Source of data: NBS and own calculation

Figure 7 shows the scatter plot of all the provinces’ cash-crop ratios (*x*-axis) versus fertilizer surpluses (*y*-axis) in China over a 3-year average from 2017 to 2019. Our results showed that the provinces with above-average fertilizer surpluses can be roughly divided into two clusters: the traditional grain cereal producers with intensive farming on the North China Plain (NCP), i.e., Shandong, Henan, Hebei, Anhui, Jiangsu, and Tianjin as well as those provinces with high cash-crop ratios in southeast and northwest China. While provinces on the NCP have either reached (G1a and 1b) or are approaching (G1c) the turning point, provinces with high cash-crop ratios still mostly show a linear growth of fertilizer surpluses when per capita GRP increases. Driven by the high returns and subsidized fertilizer prices, farmers producing cash crops normally tend to overuse fertilizers (Zhen et al. 2006; Li et al. 2013b). Nowadays, cash crops such as fruit and vegetables account for 30% of China’s total consumption of N and P fertilizers, leading to low nutrient use efficiencies and high fertilizer surpluses (Heffer 2013; Zhang et al. 2015). Although commercialized cash crop farming has brought wealth to numerous farmers, the dilemma between profitability and environmental sustainability remains.

Contrary to the majority of the provinces that exhibit a positive relationship between fertilizer surpluses and economic growth, Heilongjiang (G1c) and Jilin (G2) in northeast China have both low cash-crop ratios and low fertilizer surpluses. The EKC trajectories of the two provinces were probably influenced by the northeast region’s socio-economic conditions, in which economic recessions, population declines, and urban shrinkage have been observed in recent decades (Li 1996; Tan et al. 2017; Yang 2019).

## Conclusion

This study investigated the relationship between chemical fertilizer surpluses and economic development on the regional level in China. We employed a balanced panel dataset covering 30 provinces of mainland China from 1988 to 2019. Unit root tests, such as the ADF, PP, and KPSS tests, were conducted to determine the variables’ order of integration, EKC regressions were estimated using FM-OLS for cointegrating polynomial regressions, and group-mean coefficients were computed for each zone. Our results suggested that all of the provinces exhibit a long-run cointegrated relationship between fertilizer surpluses and per capita real GRP. A total of 22 out of 30 provinces showed a significant inverted U-shaped EKC between fertilizer surpluses and economic growth. Among the 22 provinces, 8 provinces are considered to have passed the peak, indicating that the fertilizer surpluses have been decreasing as per capita GRP grows. These provinces include Shandong, Jiangsu, Jiangxi, Hubei, Hunan, Qinghai, Sichuan, and Guizhou, and the average per capita real GRP at the peak is CNY 4044. Six provinces are considered to be at the peak, transitioning to a phase of declining environmental degradation when the economy grows. Those provinces are Liaoning, Shanxi, Anhui, Gansu, Ningxia, and Tibet, with an average per capita GRP at the peak CNY 4890. A total of 8 provinces are considered to be before the peak, meaning their fertilizer surpluses are still increasing while the economy grows, with diminishing increasing rates. These provinces include Yunnan, Heilongjiang, Henan, Tianjin, Hebei, Inner Mongolia, Shaanxi, and Xinjiang, with the average peak GRP of CNY 9820 per capita. The group-mean panel results showed that the regional mean turning points are CNY 7022, CNY 9726, CNY 4697, CNY 3749, and CNY 5588 per capita real GRP for Northeast, Northcentral, Middle, and lower reaches of the Yangtze River, and Southwest and Northwest China, respectively. The overall turning point for China is CNY 6705 per capita real GDP. This was reached in circa 2012.

Our results provide more empirical evidence that the gradual implementation of the “Zero growth plan for fertilizers by 2020” since 2015 has improved the overall fertilizer management in China. While several studies have evaluated the execution of the Zero growth plan in terms of absolute reduction of the fertilizer consumption (e.g., Jin et al. 2019), we are the first to have considered the impact of the policy in the framework of the EKC. Our findings show that multiple provinces’ turning points on the EKC were likely pulled to lower positions due to the recent decline in fertilizer consumption. This revealed the positive impacts of effective policies on the environmental performance of agricultural production. If appropriate policies are in place and effectively executed, then economic growth and environmental sustainability could be compatible.

On the basis of our empirical results, we propose the following policy recommendations. (1) Despite the existing agrochemical related policies, continuous efforts are needed to further reduce chemical fertilizer consumption in China. Supervision and agricultural extension services should be enhanced in order to promote scientific fertilization concepts to the farmers. (2) Incentives and guidelines for the proper use of organic fertilizers should be provided to farmers in order to increase the share of organic fertilization and to close the nutrient cycle. (3) Special attention on fertilizer use reduction should be paid to regions with high cash-crop ratios. This especially refers to the provinces in Southeast China, where Guangdong, Fujian, Hainan, Guangxi, and Zhejiang still exhibited linear growth in fertilizer surpluses when the economy grows. At the same time, nutrient use efficiencies should be improved, especially for cash crops such as vegetables and tropical fruit.

Small sample size and data unavailability were the major limiting factors in this study. The former increased the uncertainty in our statistical analysis, since both the unit root tests and cointegration tests are known to suffer from size distortions and a lack of power in small samples. And the latter made it difficult to include other potentially intervenient variables that would satisfy all panels. As part of the future scope of the study, country-specific causal relationship between fertilizer-related policies and the shapes and turning points of the EKC can be investigated. Moreover, structural equation models can be built and applied in the context of fertilizer surpluses-economic growth nexus. This would help to keep up with the increasing complexity of this research topic.

## Data Availability

The datasets used and analyzed during the current study are available from the corresponding author on reasonable request.

## Appendix

**Table 7:** A summary of the stationarity tests of the variables at level and after the first differencing. The numbers in the column refer to provinces for which the respective null hypothesis was rejected at the 5% significance level (*T* = 30). More detailed results of the unit root tests can be obtained from the authors upon request

	ADF test^a^		PP test		KPSS test	
	Constant	Trend	Constant	Trend	Constant	Trend
NPsur	12	6	16	1	28	26
GRP	3	4	1	1	30	12
Δ NPsur	12	–	27	–	25	–
Δ GRP	13	–	25	–	17	–

^a^A maximum number of one lag was used in the ADF test, and the optimal lag length was chosen based on the AIC

**Table 8:** Coefficient estimations from the robustness check with a control variable

	**FM-OLS estimates**			
**Province**	${\beta }_{0}$	${\beta }_{1}$	${\beta }_{2}$	$\gamma$
Liaoning	5.03***	0.7***	− 0.15***	0.32***
Jilin	4.77***	0.36***	− 0.04	0.29**
Heilongjiang	3.93***	0.74***	− 0.27***	0.24***
Beijing	3.62***	0.55***	− 0.17***	− 0.34***
Tianjin	4.1***	2.32***	− 0.32***	0.6**
Hebei	5.05***	0.67***	− 0.14***	0.24**
Shanxi	4.83***	0.59***	− 0.15***	0.13
Shandong	5.07***	0.66***	− 0.18***	0.13
Henan	5.02***	0.52***	− 0.13***	0.02
Shanghai^a^	7.92***	− 0.18	0.21	0.79**
Jiangsu	5.1***	0.68***	− 0.18***	0.07
Zhejiang^a^	4.51***	0.04	− 0.001*	− 0.21
Anhui	5.24***	0.54***	− 0.16***	0.21
Jiangxi	4.68***	0.38***	− 0.15***	0.07
Hubei	5.66***	1***	− 0.27***	0.47***
Hunan	4.65***	0.34***	− 0.12***	− 0.01
Fujian^a^	4.79***	0.11	0.03	− 0.17
Guangdong^a^	4.77***	− 0.26	0.1	− 0.23
Guangxi	4.84***	0.39***	− 0.05*	0.09
Hainan	4.37***	0.72***	− 0.11	− 0.2
Sichuan	4.88***	0.36***	− 0.12***	0.06
Guizhou	4.83***	0.31***	− 0.21***	0.16
Yunnan	4.25***	0.31***	− 0.1***	− 0.29*
Tibet	4.1***	0.64***	− 0.27***	− 0.17
Inner Mongolia	4.29***	0.71***	− 0.11***	0.18*
Shaanxi	5.85***	1.14***	− 0.27***	0.7**
Gansu	5.11***	0.73***	− 0.27***	0.4
Qinghai	4.83***	0.77***	− 0.27***	0.28**
Ningxia	5.72***	0.99***	− 0.28***	0.69**
Xinjiang	4.79***	1.16***	− 0.29***	0.5***

^a^Since the linear terms of Shanghai, Zhejiang, Fujian, and Guangdong were insignificant in the quadratic model specification, we re-estimated the linear model specification for those four provinces. The *t*-statistics showed that the linear terms were still insignificant in the linear model specification

**Table 9:** A comparison of results from the reduced model (**Eq. ****2****)** and the model with control variable (**Eq. ****3****)**

	**Reduced model (Eq. ** **2** **)**	**Model with control variable (Eq. ** **3** **)**
**Inverted U-shaped curve** (*p*_*β*_2_ < 0.05 and *β*_2_ < 0)	**(22)** Liaoning, Heilongjiang, Tianjin, Hebei, Shanxi, Shandong, Henan, Jiangsu, Anhui, Jiangxi, Hubei, Hunan, Sichuan, Guizhou, Yunnan Tibet, Inner Mongolia, Shaanxi, Gansu, Qinghai, Ningxia, Xinjiang	**(23)** Liaoning, Heilongjiang, Beijing, Tianjin, Hebei, Shanxi, Shandong, Henan, Jiangsu, Anhui, Jiangxi, Hubei, Hunan, Sichuan, Guizhou, Yunnan, Tibet, Inner Mongolia, Shaanxi, Gansu, Qinghai, Ningxia, Xinjiang
**Linear increase** (*p*_*β*_2_ > 0.05, *p*_*β*_1_ < 0.05, and *β*_1_ > 0)	**(7)** Beijing, Jilin, Guangxi, Hainan, Guangdong, Zhejiang, Fujian	**(3)** Jilin, Guangxi, Hainan; ***(2*** ^***a***^ ***)*** *Zhejiang (insignificant), Fujian (insignificant)*
**Linear decrease** (*p*_*β*_2_ > 0.05, *p*_*β*_1_ < 0.05, and *β*_1_ < 0)	**(1)** Shanghai	***(2*** ^***a***^ ***)*** *Shanghai (insignificant), Guangdong (insignificant)*

^a^Signs of the coefficient match with the criteria, but not the significance level
